# What role does the seed coat play during symbiotic seed germination in orchids: an experimental approach with *Dendrobium officinale*

**DOI:** 10.1186/s12870-022-03760-0

**Published:** 2022-07-29

**Authors:** Xiang-Gui Chen, Yi-Hua Wu, Neng-Qi Li, Jiang-Yun Gao

**Affiliations:** Institute of Biodiversity, School of Ecology and Environmental Science, Yunnan University, Kunming, 650500 Yunnan China

**Keywords:** Compatible fungi, Incompatible fungi, Orchid mycorrhizal fungi, Plant-fungus interactions, Symbiotic seed germination, Dust seeds

## Abstract

**Background:**

Orchids require specific mycorrhizal associations for seed germination. During symbiotic germination, the seed coat is the first point of fungal attachment, and whether the seed coat plays a role in the identification of compatible and incompatible fungi is unclear. Here, we compared the effects of compatible and incompatible fungi on seed germination, protocorm formation, seedling development, and colonization patterns in *Dendrobium officinale*; additionally, two experimental approaches, seeds pretreated with NaClO to change the permeability of the seed coat and fungi incubated with in vitro-produced protocorms, were used to assess the role of seed coat played during symbiotic seed germination.

**Results:**

The two compatible fungi, *Tulasnella* sp. TPYD-2 and *Serendipita indica* PI could quickly promote *D. officinale* seed germination to the seedling stage. Sixty-two days after incubation, 67.8 ± 5.23% of seeds developed into seedlings with two leaves in the PI treatment, which was significantly higher than that in the TPYD-2 treatment (37.1 ± 3.55%), and massive pelotons formed inside the basal cells of the protocorm or seedlings in both compatible fungi treatments. In contrast, the incompatible fungus *Tulasnella* sp. FDd1 did not promote seed germination up to seedlings at 62 days after incubation, and only a few pelotons were occasionally observed inside the protocorms. NaClO seed pretreatment improved seed germination under all three fungal treatments but did not improve seed colonization or promote seedling formation by incompatible fungi. Without the seed coat barrier, the colonization of in vitro-produced protocorms by TPYD-2 and PI was slowed, postponing protocorm development and seedling formation compared to those in intact seeds incubated with the same fungi. Moreover, the incompatible fungus FDd1 was still unable to colonize in vitro-produced protocorms and promote seedling formation.

**Conclusions:**

Compatible fungi could quickly promote seed germination up to the seedling stage accompanied by hyphal colonization of seeds and formation of many pelotons inside cells, while incompatible fungi could not continuously colonize seeds and form enough protocorms to support *D. officinale* seedling development. The improvement of seed germination by seed pretreatment may result from improving the seed coat hydrophilicity and permeability, but seed pretreatment cannot change the compatibility of a fungus with an orchid. Without a seed coat, the incompatible fungus FDd1 still cannot colonize in vitro-produced protocorms or support seedling development. These results suggest that seed coats are not involved in symbiotic germination in *D. officinale*.

**Supplementary Information:**

The online version contains supplementary material available at 10.1186/s12870-022-03760-0.

## Background

Mycorrhiza, the symbiotic association between plant roots and fungi, is commonly found in the majority of vascular plants and includes four main types: arbuscular mycorrhiza (AM), ectomycorrhiza (EcM), ericoid mycorrhiza (ErM) and orchid mycorrhiza (OM) based on the criteria of morphological differentiation of root tissues and host plant lineages [1]. Mycorrhizal fungi (MFs) benefit most plants by enhancing their nutrient access and stress tolerance and therefore strongly affect plant population and community biology by regulating seedling establishment and species coexistence [2]. This symbiotic association is particularly important for orchids because most orchids rely completely on mycorrhizal fungi for seed germination [3, 4].

Orchids produce large numbers of dust-like seeds, which are characterized by a small and undeveloped embryo not surrounded by an endosperm [5]; therefore, orchid seeds require specific mycorrhizal fungi for mineral and carbon resources to germinate into seedlings under natural conditions [3, 4]. Orchid mycorrhizal fungi (OMFs) are recruited from rhizoctonias that are often considered to live as saprobes in soil around the roots or on tree bark around epiphytic orchids [3, 6]. Therefore, host-fungus compatibility might be influenced largely by environmental factors [3]. An increasing number of studies have shown that in the seed germination stage, orchids require more specific mycorrhizal associations, and incompatible fungi may stimulate germination per se but not support subsequent seedling development [7–14]. One interesting question during seed symbiotic germination is how the interaction is established between an orchid and its fungal partners [15].

In AM association, the gene expression and signalling pathways during root colonization have been well studied. The recognition of symbiotic signalling molecules allows host plants to quickly differentiate compatible symbionts from the greater population of microbes, while mycorrhizal factors secreted from AM fungi have been identified as key signals used to communicate with host plants, allowing the entry of the microbial partners into plant cells [16]. However, it is still unclear whether the molecular interactions, compounds and signalling pathways involved in AM are similar in OM or if there is another specific OM establishment mechanism [17]. Moreover, the OM association involves fungi colonizing roots and seeds.

Orchid seeds are among the smallest seeds among all flowering plants. Although the macroscopic appearances of different orchid seeds are similar, the seeds are highly diverse owing to their seed coats [5, 18]. In most orchid species, the seed coat usually consists of a transparent and single-cell layer and encloses an embryo with a distinctive feature of a large internal airspace between the embryo and seed coat [5, 19], which is considered to be related to the dispersal strategy, seed dormancy, and water uptake and storage [5, 20, 21]. During seed symbiotic germination, fungal hyphae grow into orchid tissues through the suspensor cells of seeds and form pelotons within cortical cells [22, 23]. Compatible fungi can persistently colonize orchid seeds and quickly promote the conversion of germinated seeds into seedlings, while incompatible fungi do not persistently colonize seeds or further support seedling development [24].

In orchid asymbiotic seed germination (in vitro seed germination on medium without fungal symbionts), seed pretreatments with NaClO solution are commonly used to increase the hydrophilic character of the seed coat and make the seed coats more permeable, significantly improving seed germination [25, 26]. Although the pathways of nutrient uptake are completely different between asymbiotic and symbiotic germination, germinated seeds all form protocorms that are rootless and acotyledonous conical to spherical bodies and known as undifferentiated seedlings or the first seedling stage of orchids [27]. New leaves and roots are formed successively from the meristematic domain at the anterior end of the protocorm [28]. Since orchid seeds are so tiny and simply structured and seed coats are the first point of fungal attachment during symbiotic germination, an interesting question is what roles do the seed coats play during symbiotic seed germination? By removing the seed coat of *Bletilla striata* using a dissecting needle under a stereomicroscope, it was found that the seed coat-stripped seeds inoculated with the symbiotic fungi showed a lower germination rate than the intact seeds, and inoculation with pathogenic fungi resulted in an increased infection rate in the seed coat-stripped seeds, suggesting that the seed coat restricts the invasion of fungal hyphae and protects the embryo against attack from nonsymbiotic fungi [29].

To better understand the roles of the seed coat during symbiotic seed germination, in this study, by using a well-studied medicinal orchid *Dendrobium officinale*, we compared the effects of compatible and incompatible fungi on seed germination and their colonization patterns and then tested the effects of seed pretreatments on seed germination and seedling formation in symbiotic germination and whether fungi could easily colonize in vitro-produced protocorms (without seed coats) and promote seedling formation. Here, we present our results, addressing three principal questions: (1) Do the effects of different fungi on seed germination match their colonization patterns? (2) Can seed pretreatments improve seed germination and seedling formation in symbiotic germination as the results of improvement of seed coat permeability, and, in particular, promote seed colonization by incompatible fungi? (3) Do compatible and incompatible fungi effectively colonize in vitro-produced protocorms and promote seedling formation?

## Results

### Seed incubation with different mycorrhizal fungi

In this experiment, five seed incubation treatments were conducted including three fungal treatments (two compatible fungi TPYD-2 and PI and one incompatible fungi FDd1) and two control treatments without a fungal strain on OMA medium (nutrient poor-medium) and MS medium (nutrient rich-medium). In all three fungal treatments and the MS treatment, seeds of *D. officinale* started to germinate (Stage 1) at 12 days after incubation. Subsequently, until 22 days after incubation, numbers of germinated seeds increased rapidly and formed large numbers of protocorms (mainly at Stage 2) in the two compatible fungal treatments and MS treatment, but no protocorms were found in the incompatible fungal FDd1 treatment and OMA treatment. The percentage of protocorms in the TPYD-2 treatment was 66.4 ± 2.40%, which was significantly higher than that in the PI (48.7 ± 2.04%) and MS treatments (47.9 ± 1.59%) (all *P* < 0.05).

**Fig. 1:**
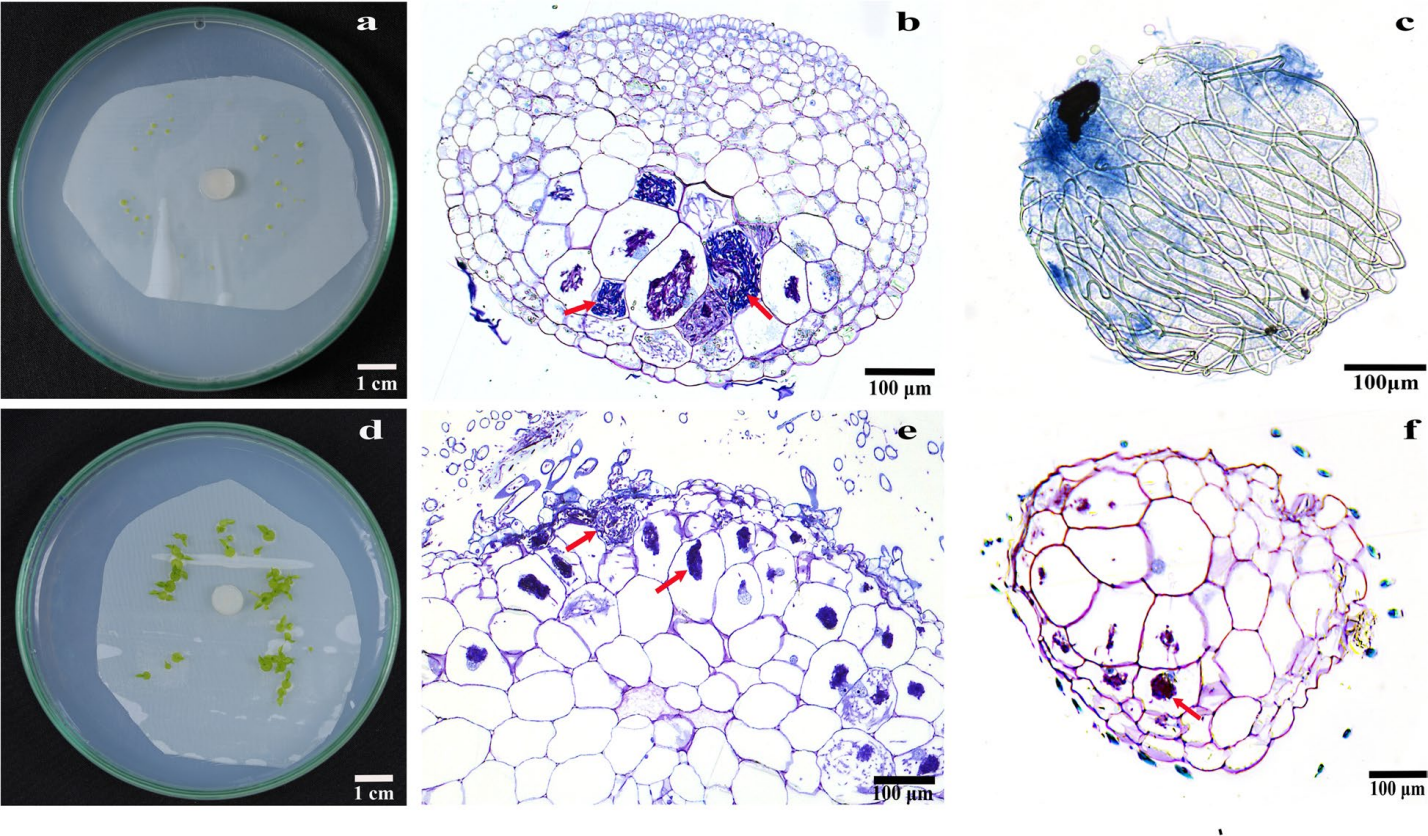
The status of seed germination, fungal hyphae on seeds, and pelotons inside protocorms or seedlings at 32 and 62 days after the incubation of seeds with compatible or incompatible fungal strains in *Dendrobium officinale*. **a**, Protocorms (Stage 2 or 3) in two compatible fungal treatments at 32 days after incubation; **b**, Cross-sections of protocorm showing pelotons in the basal cells in two compatible fungal treatments at 32 days after incubation; **c**, Fungal hyphae clustered on the seed surfaces under the incompatible fungal FDd1 treatment at 32 days after incubation; **d**, Seedlings in the compatible PI treatment bore two leaves (Stage 5) and some had one or two roots at 62 days after incubation; **e**, Cross-sections of seedling showing massive pelotons inside basal cells in two compatible fungal treatments at 62 days after incubation; **f**, Cross-sections of protocorm showing pelotons occasionally observed in the basal cells in the incompatible fungal FDd1 treatment at 62 days after incubation. The red arrows indicate pelotons inside protocorm or seedling cells

Ten days later, at 32 days after incubation, most of the seeds had already formed protocorms (Stages 2 or 3) in the two compatible fungal treatments (Fig. 1a). The percentages of protocorms in the TPYD-2 treatment reached 77.3 ± 1.71%, which was significantly higher than that in the PI treatment (71.6 ± 2.53%; *P* < 0.05), but both values were significantly higher than that in the MS treatment (57.0 ± 1.70%; all *P* < 0.001). No seedlings were found in all treatments. Seeds were still at Stage 1, and no protocorms occurred in either the FDd1 treatment or OMA treatment. At this stage, fungal hyphae could be observed growing into seeds through suspensors and formed many pelotons in the basal cells of the protocorm in both compatible fungal treatments (Fig. 1b). In the incompatible fungal FDd1 treatment, some fungal hyphae could also be observed congregated around the suspensor at the basal end outside of the seeds (Fig. 1c).

**Fig. 2:**
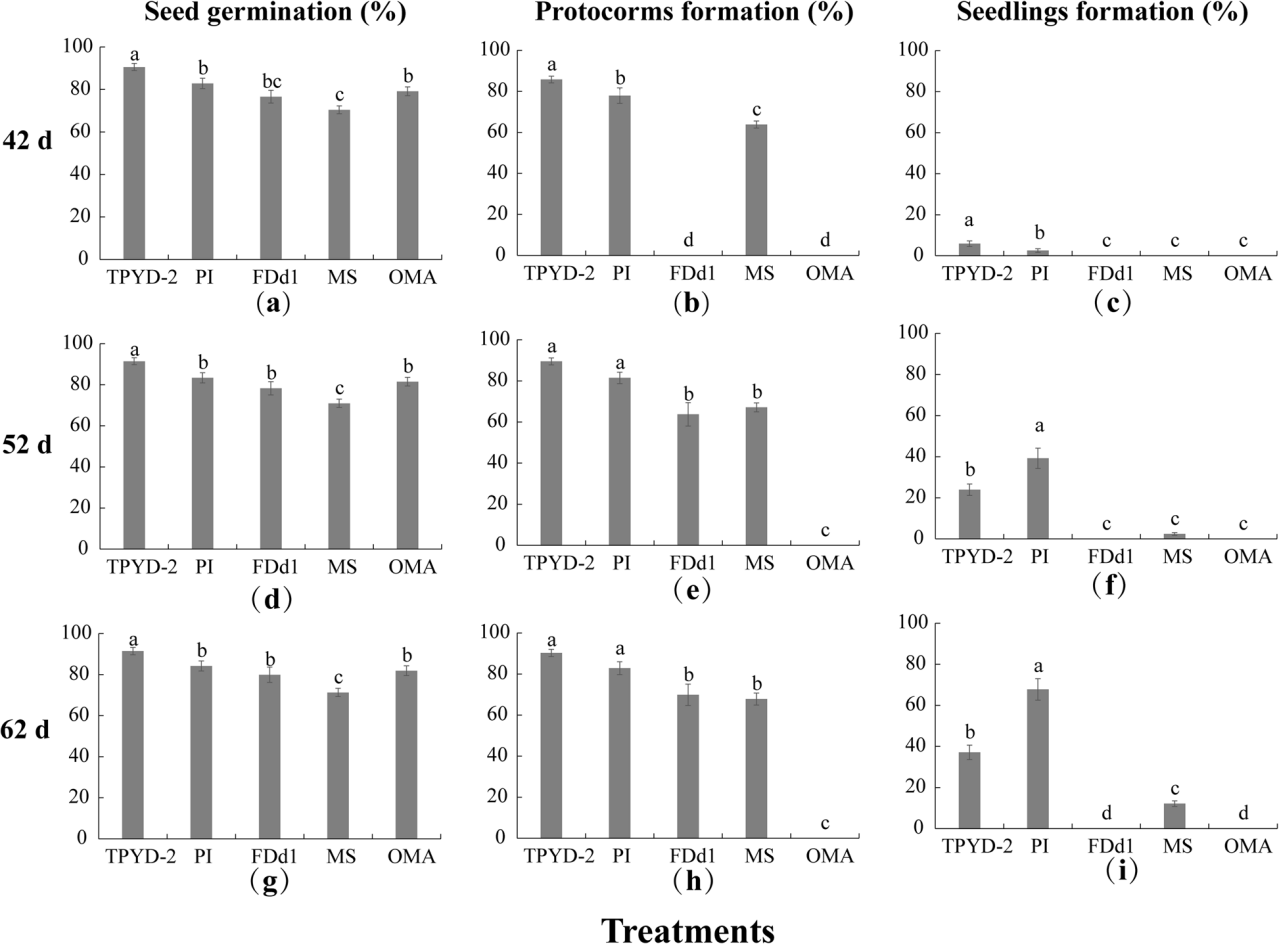
Effects of different treatments (seeds on OMA and MS and seeds on OMA with addition of the fungi TPYD-2, PI or FDd1) on seed germination, protocorm formation and seedling development at 42, 52 and 62 days after incubation in *Dendrobium officinale*. In each panel, different letters indicate significant differences (*p* < 0.05) based on one-way ANOVA and the least significant difference (*LSD*) method where the data are normally distributed and the generalized linear model (*GLM*) where the data are not normally distributed

At 42 days after incubation, a few seedlings at the first leaf stage (Stage 4) occurred in both the compatible fungal TPYD-2 treatment (5.9 ± 1.34%) and the PI treatment (2.5 ± 0.91%). The percentages of protocorms in the TPYD-2 treatment (85.8 ± 1.65%) were significantly higher than those in the PI treatment (77.9 ± 3.75%; *P* < 0.05) and in the MS treatment (63.8 ± 1.74%; *P* < 0.001). Although most of the seeds had germinated (Stage 1) in the incompatible fungal FDd1 treatment (76.6 ± 0.00%) and OMA treatment (79.2 ± 2.07%), no protocorms were found in either treatment (Fig. 2).

At 52 days after incubation, there was no significant difference in the percentages of protocorms between the two compatible fungal treatments (TPYD-2: 89.5 ± 1.73% *vs*. PI: 81.5 ± 2.78%; *P* = 0.054), but the percentages of seedlings in the PI treatment (39.2 ± 4.98%) were significantly higher than those in the TPYD-2 treatment (23.9 ± 2.81%; *P* < 0.001). At this stage, a large number of protocorms (Stage 2) formed in the incompatible fungal FDd1 treatment (63.8 ± 5.71%), and a few seedlings (2.4 ± 0.69%) were also found in the MS treatment (Fig. 2).

At 62 days after incubation, number of seedlings in the PI treatment increased greatly compared with 10 days ago, and most seedlings bore two leaves (Stage 5), while some seedlings (8.0 ± 7.74%) had grown one or two roots (Fig. 1d). The percentage of seedlings in the PI treatment (67.8 ± 5.23%) was significantly higher than that in the TPYD-2 treatment (37.1 ± 3.55%; *P* < 0.001). For the incompatible fungus FDd1 treatment, protocorms remained unchanged (Stage 2), and no seedlings were found. In the two control treatments, some seedlings occurred in the MS treatment (12.1 ± 1.35%), but all seeds were still in Stage 1 and no protocorms were found in the OMA treatments (Fig. 2). At this stage, massive pelotons occurred inside the basal cells of the protocorm or seedlings in both compatible fungal treatments (Fig. 1e), and some pelotons were also occasionally observed in the basal cells of the protocorm in the incompatible fungal FDd1 treatment (Fig. 1f).

### Incubation of pretreated seeds with different mycorrhizal fungi

Overall, short-term seed pretreatment (e.g., 5 and 10 min) had positive effects on seed germination, protocorm formation and seedling development, while longer-term seed pretreatment (e.g., 20, 30 and 40 min) showed different effects on seed germination, protocorm formation and seedling development among the three different fungal treatments. At 52 days after incubation, no seedlings were formed in any of the treatments of pretreated seeds incubated with the incompatible fungus FDd1 (Additional File 1 Table S1). For the two compatible fungal treatments, seed pretreatment obviously promoted seed germination, while fungi showed strong effects on protocorm formation and seedling development, as indicated by the *F* values given by two-way ANOVA (Table 1).

**Table 1:** The results of two-way ANOVA showing the *F* values and *P* values for the effects of seed pretreatment time and fungi on seed germination, protocorm formation and seedling development in *Dendrobium officinale*

Source of variation	Dependent variable	d.f	Mean square	*F Value*	*P Value*
Seed pretreatment time	Seed germination	5	6392.968	31.992	<0.001
	Protocorm formation	5	5654.608	17.275	<0.001
	Seedling development	5	3465.742	8.732	<0.001
Fungus	Seed germination	2	3939.513	19.714	<0.001
	Protocorm formation	2	8967.404	27.395	<0.001
	Seedling development	2	132,467.565	333.769	<0.001
Seed pretreatment time * Fungus	Seed germination	10	504.311	2.524	0.06
	Protocorm formation	10	1286.202	3.929	<0.001
	Seedling development	10	2204.718	5.555	<0.001

**Fig. 3:**
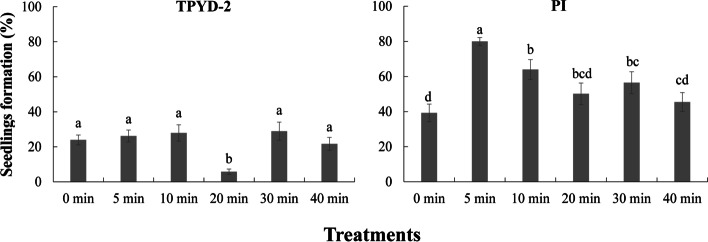
Effects on seedling development from seeds pretreated with 1% (w/v) NaClO for 5, 10, 20, 30 and 40 min under two compatible fungi (TPYD-2 and PI) treatments. The significant differences among seed pretreatment durations in the two fungal treatments are shown by different lowercase letters at *p* < 0.05

However, seed pretreatment time did not significantly increase the percentages of seedlings in TPYD-2 treatments, and the percentages of seedlings in the 20-min seed pretreatment abnormally fell to 5.8 ± 1.51%, which was significantly lower than that in other treatments (Fig. 3). Compared to the control treatment, seed pretreatment obviously promoted seedling development in the PI treatment, and the percentage of seedlings in the 5-min seed pretreatment (80.0 ± 2.21%) was significantly higher than that in any other treatment (all *P* < 0.05; Fig. 3).

### Protocorms incubation with different mycorrhizal fungi

**Fig. 4:**
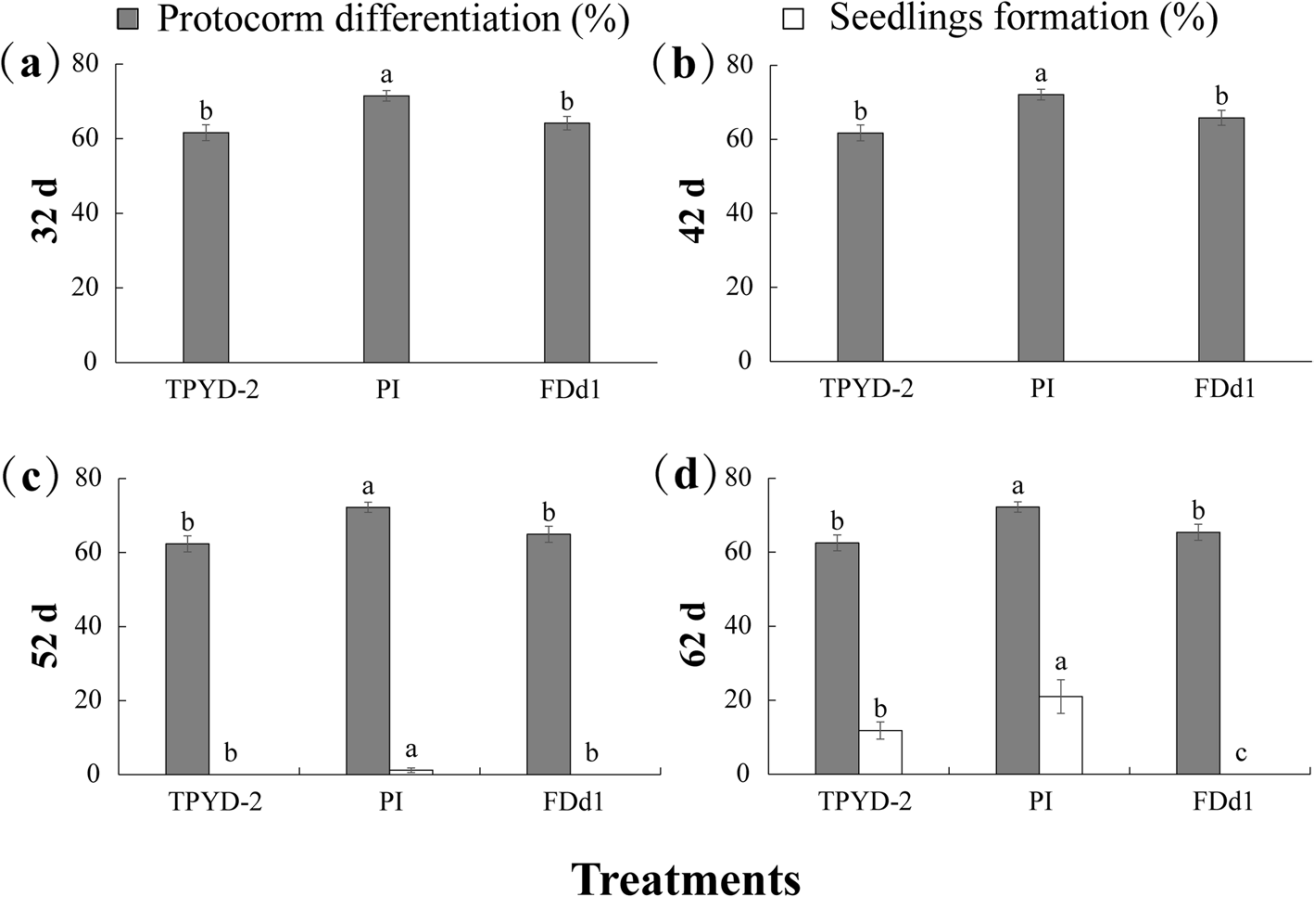
Effects of in vitro-produced protocorms incubated with different mycorrhizal fungi (TPYD-2, PI or FDd1) on protocorm differentiation and seedling formation at 32, 42, 52 and 62 days after incubation in *Dendrobium officinale*. The significant differences among fungal treatments are shown by different lowercase letters at *p* < 0.05. The protocorms were obtained by in vitro-seed germination on MS medium for 22 days to Stage 2

After transfer to OMA medium, the tiny protocorms (Stage 2; Additional File 2 Fig. S1) slowly and continuously developed into large protocorms (Stage 3) in all treatments. At 32 days after incubation, most protocorms reached Stage 3 (Fig. 4a); however, subsequently, protocorms appeared to remain unchanged, and the percentages of protocorms at Stage 3 did not increase at 42 days after incubation compared with those 10 days ago in all fungal treatments (Fig. 4b). At 52 days after incubation, very few seedlings appeared in the PI treatment (1.2 ± 0.64%), and then seedlings increased continuously in the two compatible fungal treatments (Fig. 4c).

**Fig. 5:**
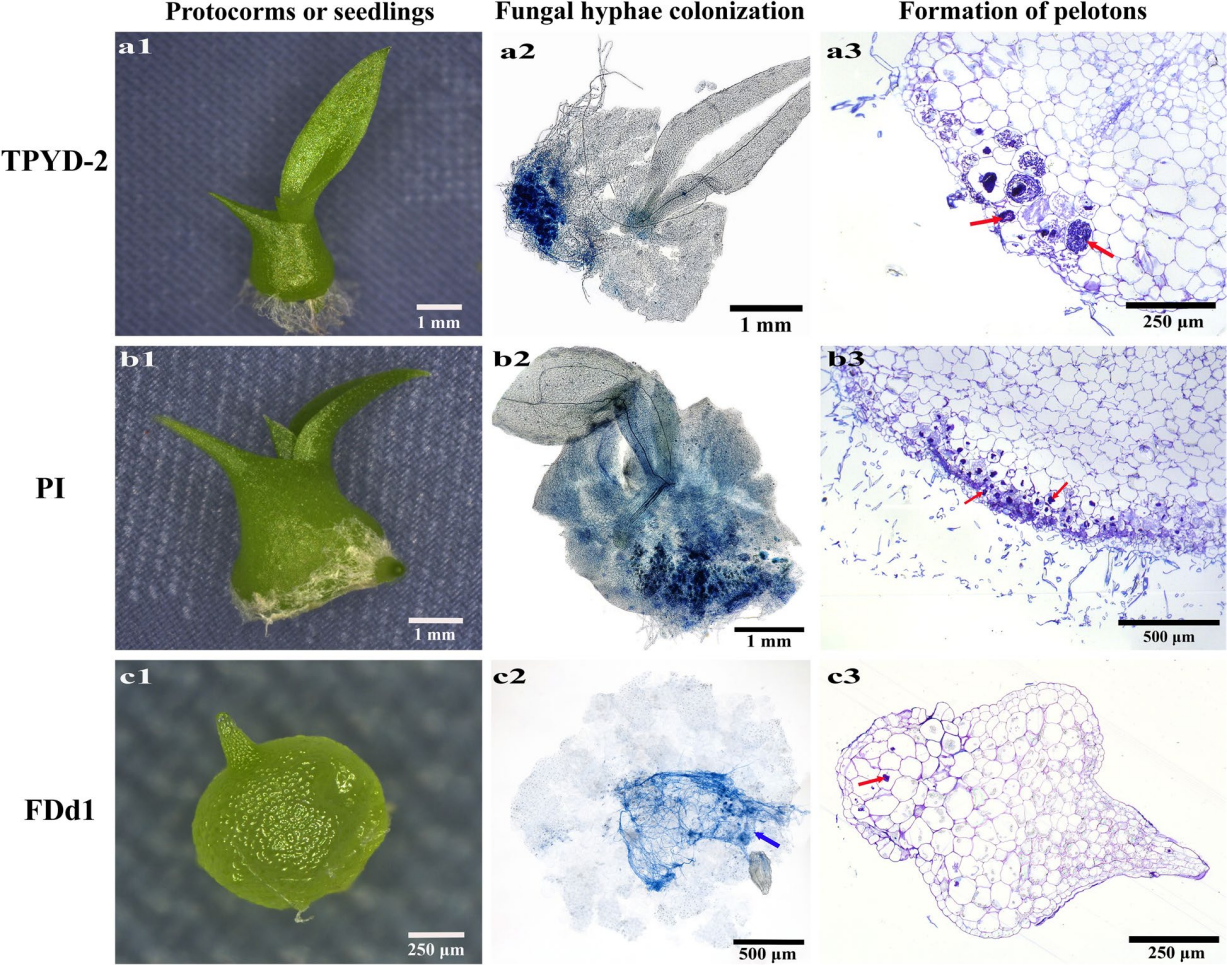
Protocorm or seedling, status of fungal hyphae colonization, and cross-sections of protocorms and seedlings showing pelotons under the treatments of in vitro-produced protocorms incubated with three different mycorrhizal fungi at 62 days after incubation. (a1-a3) Compatible fungus TPYD-2 treatment; (b1-b3) compatible fungus PI treatment; (c1-c3) incompatible fungus FDd1 treatment. The red arrows indicate pelotons inside the cells of protocorms or seedlings

At 62 days after incubation, the percentage of seedlings in the PI treatment was 21.0 ± 4.57%, which was significantly higher than that in the TPYD-2 treatment (11.8 ± 2.32%; *P* < 0.05), while no seedlings were found in the incompatible fungal FDd1 treatment (Fig. 4d). At this stage, seedlings with one leaf (Stage 4) were common in the TPYD-2 treatment (Fig. 5a1), and fungal hyphae colonization (Fig. 5a2) at the bases of the protocorm and many pelotons inside the cells of the protocorm (Fig. 5a3) could be observed. Most seedlings in the PI treatment bore 3–4 leaves (Stage 5), some had one newly developed root (Fig. 5b1), and a large number of fungal hyphae colonized the protocorm (Fig. 5b2) and formed pelotons (Fig. 5b3). However, in the incompatible fungal FDd1 treatment, protocorms still had not differentiated into seedlings (Fig. 5c1), fungal hyphae congregated at the basal end outside of protocorms (Fig. 5c2), and only a few pelotons inside the protocorm cells were occasionally observed (Fig. 5c3).

## Discussion

In orchids, seed germination and seedling establishment are considered the key processes for the establishment, distribution, abundance and dynamics of populations, which are largely affected by symbioses with mycorrhizal fungi [30–32]. OMFs are considered to originate from fungal ancestors colonizing roots as endophytes [33] and are now known to belong to a polyphyletic group of rhizoctonia, including fungi from Tulasnellaceae, Ceratobasidiaceae and Serendipitaceae [3, 34, 35]. These taxa are not mycorrhizal in other plants and are often considered to live as saprobes in soil around the roots or on tree bark around epiphytic orchids [3, 6]. Therefore, host-fungal compatibility may be influenced largely by environmental factors [3].

*Dendrobium officinale* is a widely distributed orchid with diverse habitats, and a wide range of fungi originating from different habitats/resources, many of which are able to promote seed germination and seedling development with relative effectiveness, have been reported to associate with *D. officinale* [14, 36–39]. In this study, three mycorrhizal fungi were used to compare their effects on *D. officinale* seed germination*.* The two compatible fungi, *Tulasnella* sp. TPYD-2 and *Serendipita indica* PI could quickly promote seed germination up to the seedling stage with relative effectiveness, and two fungi persistently colonized seeds and continuously formed a large number of pelotons inside the cells. In both compatible fungal treatments, as well as the MS treatment, seeds of *D. officinale* started to germinate (Stage 1) at 12 days after incubation, formed large numbers of protocorms (mainly at Stage 2) at 22 days after incubation and developed seedlings (Stage 4) at 42 days after incubation, but the percentages of seed germination, protocorms and seedlings at different stages varied greatly among the three treatments, suggesting that two compatible fungi, *Tulasnella* sp. TPYD-2 and *Serendipita indica* PI had different effects on seed germination, protocorm formation and seedling development in *D. officinale*, but all performed much better in both fungal treatments than in the asymbiotic germination treatment (MS treatment).

In contrast, in the incompatible fungus *Tulasnella* sp. FDd1 treatment, seeds started to germinate (Stage 1) at 42 days after incubation and formed protocorms (Stage 2) 52 days after incubation; moreover, no seedlings developed until 62 days after incubation. Although hyphae were observed congregated at the basal end outside of seeds at the beginning of fungal incubation, FDd1 could not persistently colonize seeds of *D. officinale* and form enough protocorms to support seedling development. These results strongly suggested that the pattern of hyphal colonization of seeds was well matched with the morphological differentiation of seed germination and seedling development, and compatible fungi could persistently colonize seeds and quickly promote the conversion of germinated seeds into seedlings [24].

In the seed pretreatment experiment, as expected, seeds pretreated with NaClO showed improved germination in all three fungal treatments, including the incompatible fungal FDd1 treatment, and relatively positive effects on protocorm formation and seedling development were also shown in the two compatible fungal treatments. However, seed pretreatment could not “help” the incompatible fungus FDd1 colonize seeds, as indicated by the absence of seedling formation in all treatments of pretreated seeds incubated with FDd1. That is, seed pretreatment cannot change the compatibility of a fungus with an orchid. The improvement of seed germination, protocorm formation and seedling development may result from the improvement of the hydrophilic character and permeability of the seed coat, as confirmed in asymbiotic germination of different orchid species [25, 26]. The results of statistical analysis also confirmed that seed pretreatment significantly promoted seed germination, while fungi showed strong effects on protocorm formation and seedling development (Table 1).

After incubation with fungi, the development of in vitro-produced protocorms was greatly slowed compared to that under the treatments of intact seeds incubated with the same fungi. In the PI treatment, only a few seedlings (1.2 ± 0.64%) appeared until 52 days after incubation, and the percentage of seedlings was 21.0 ± 4.57% at 62 days after incubation, while in the seed-PI treatment, seedlings occurred at 42 days after incubation, and the percentages of seedlings reached 39.2 ± 4.98% and 67.8 ± 5.23% at 52 and 62 days after incubation, respectively. The same situation also occurred in the TPYD-2 treatment; no seedlings were found until 52 days after incubation, and 11.8 ± 2.32% of protocorms developed into seedlings at 62 days after incubation, while in the seed-TPYD-2 treatment, seedlings occurred at 42 days after incubation, and the percentages of seedlings were 23.9 ± 2.81% and 37.1 ± 3.55% at 52 and 62 days after incubation, respectively. Interestingly, without the barrier of the seed coat, the incompatible fungus FDd1 was still unable to colonize protocorms and promote seedling formation. At 62 days after incubation, many fungal hyphae could be observed congregating at the basal end outside of protocorms but not entering protocorms.

During the interactions between fungi and orchid seeds, fungal hyphae grow into orchid tissues through the suspensor at the end of the seeds [23], and nutrient uptake by the embryo also occurs through the suspensor in asymbiotic germination in some orchids [40]. In the current study, we also observed that fungal hyphae grew into seeds through the suspensor and formed pelotons in the two compatible fungal treatments, but fungal hyphae congregated around the suspensor and did not enter seeds in the incompatible fungal treatments. Both seed pretreatment and fungal incubation with protocorms could not promote colonization of seeds/protocorms or formation of pelotons inside by incompatible fungi. The mycorrhizal symbioses between orchids and fungi might be influenced by inherent differences among closely related fungi, and the symbiotic mechanisms are still unclear but may involve fungal effector and plant receptor genes similar to plant–pathogen interactions [17, 41].

## Conclusions

Compatible and incompatible fungi showed different seed colonization patterns in *D. officinale*, which resulted in great differences in seed germination and seedling formation. Both compatible fungi *Tulasnella* sp. TPYD-2 and *Serendipita indica* PI could quickly promote seed germination up to the seedling stage, accompanied by fungal hyphae colonizing seeds and forming a large number of pelotons inside the cells, while the incompatible fungus *Tulasnella* sp. FDd1 could not persistently colonize seeds or form enough pelotons to support seedling development. Overall, seed pretreatment improved seed germination, protocorm formation and seedling development to different degrees but did not change the compatibility of a fungus with an orchid. Without a seed coat, the incompatible fungus *Tulasnella* sp. FDd1 still did not colonize protocorms or support seedling development. Our results suggested that seed coats were not involved in symbiotic germination.

## Methods

### Orchid species and mycorrhizal fungi

*Dendrobium officinale* is a lithophytic or epiphytic orchid widely distributed in subtropical areas, including SW Anhui, W Fujian, NW Guangxi, Sichuan, Taiwan, SE Yunnan and E Zhejiang in China. It grows in different habitats, e.g., growing on trees in forests, rocks in karst landforms and sandy conglomerates in Danxia landforms under different vegetation types [14]. As one of the most popular medicinal orchid species in China, it has received much research attention regarding its mycorrhizal symbionts, including the isolation and identification of effective symbiotic fungi for seed germination and plant growth [14, 36–39]. In this study, we conducted outcross-pollination trials on cultivated plants of *D. officinale* during March 2018 and harvested close-to-dehiscent fruits in November 2018. The species identification of *D. officinale* was made by the corresponding author JYG who has been engaged in orchid studies for over 20 years. Seeds were dried and stored in the Orchid Seed Bank of Yunnan University. Prior to each use, seeds were tested using the TTC (2,3,5-triphenyl tetrazolium chloride) method to ensure high viability (> 96%) [42].

In this study, three mycorrhizal fungal strains were used to compare their effects on the seed germination of *D. officinale.* The compatible *Tulasnella* sp. TPYD-2 (GenBank accession No. MN545849) was isolated from seedling roots of *D. officinale* and could effectively promote seed germination and seedling growth of *D. officinale* [43]. The strain of *Tulasnella* sp. TPYD-2 has been deposited in the China Center for Type Culture Collection (CCTCC No. M2020165). *Tulasnella* sp. FDd1 (GenBank accession No. KM226996) was isolated from protocorms of *D. devonianum* and could effectively promote seed germination of *D. devonianum* but is incompatible with *D. officinale* [11]. The strain of *Tulasnella* sp. FDd1 has been deposited in the China General Microbiological Culture Collection Center (CGMCC No. 9551). Another mycorrhizal fungus used in this study, *Serendipita indica* syn. *Piriformospora indica* (PI; DSM11827) was originally obtained from the Leibniz Institute DSMZ-German Collection of Microorganisms and Cell Cultures, Braunschweig, Germany. The identity has also been confirmed again by ITS sequence. *S. indica* was first isolated from rhizosphere soil of desert shrubs [44]. According to our previous studies, *S. indica* was able to effectively promote seed germination in many orchid species, including *D. officinale,* and is compatible with *D. officinale* (Xu et al., unpublished data).

### Seed incubation with different mycorrhizal fungi

Seeds of *D. officinale* were inoculated with compatible fungi (TPYD-2 and PI) and incompatible fungi (FDd1) to compare seed germination and the morphological differences in fungal hyphae colonizing seeds during the process of symbiosis. Seeds were sterilized with 1% (w/v) NaClO for 3 min and washed with sterile distilled water 3–5 times. Surface sterilized seeds were sown on OMA (*ca*. 40 seeds per Petri dish) and inoculated with the fungi TPYD-2, FDd1 and PI following our previous methods [8]. Two control treatments without a fungal strain were carried out on OMA medium (nutrient-poor medium) and MS medium (nutrient-rich medium). Each treatment was replicated in 60 Petri dishes that were placed in illumination incubators (RXZ300B, Ningbo Southeast Instrument Co., Ltd., Ningbo, China) under the conditions of 26 ± 2.0 °C and a 12 h/12 h light/dark cycle.

The stages of seed germination for each treatment were monitored and recorded regularly. Once seed germination was observed in any treatment, 30 Petri dishes for each treatment were used to count the percentages of germinated seeds, protocorms and seedlings every 10 days until any given treatment showed mostly seedlings. Each time, another 30 Petri dishes for each treatment were used to sample the seed materials that accurately represented the most common stage in the treatment group (e.g., ungerminated seeds, germinated seeds, protocorms or seedlings). Sampled seed materials were stained following the methods described by Phillips and Hayman [45], and then the statuses of fungal hyphae colonized with seed materials were directly observed under a Leica Microsystems (DM2000, Leica Microsystems GmbH, Wetzlar, Germany). Additionally, sampled seed materials were also embedded in LR white resin, and semithin sections were stained with 1% (w/v) toluidine blue for observation of fungal hyphae colonization and pelotons inside cells.

### Incubation of pretreated seeds with different mycorrhizal fungi

Seeds of *D. officinale* were pretreated with 1% (w/v) NaClO for 5, 10, 20, 30 and 40 min and then inoculated with two compatible fungi (TPYD-2 and PI) and the incompatible fungus FDd1. A total of 15 treatments (3 fungi * 5 seed pretreatments) were conducted. Each treatment was replicated in 30 Petri dishes, and the incubation conditions were the same as those used for the above seed incubation. At 52 days after incubation, the percentages of germinated seeds, protocorms and seedlings were counted for each treatment, and seed materials were also sampled for observations of fungal hyphae colonization. We used the same date of three fungal treatments at 52 days after incubation in the above seed incubation experiment (nonpretreated seeds) as a control treatment to assess the effects of different seed pretreatments on seed germination, protocorm formation and seedling development.

### Protocorms incubation with different mycorrhizal fungi

Seeds of *D. officinale* were germinated in vitro in MS medium for 22 days to obtain protocorms (Stage 2; Additional File 2: Fig. S1). Forty protocorms were carefully transferred to a Petri dish with 20 mL OMA medium in a superclean bench, and then each Petri dish was inoculated with 1 cm^3^ of fungal inocula placed in the centre of the Petri dish for each of the TPYD-2, FDd1 and PI treatments. Each treatment was replicated in 60 Petri dishes, and the incubation conditions were the same as those used for the above seed incubation. The status of protocorm development and seedling formation for each treatment were monitored and recorded regularly. At 32, 42, 52 and 62 days after incubation, 30 Petri dishes for each treatment were used to count the percentages of protocorm development and seedling formation, and another 30 Petri dishes for each treatment were used to sample protocorm materials for observations of fungal hyphae colonization using the same method of semithin sections described above.

### Data collection and statistical analysis

Orchid seed germination was divided into five stages following the criterion proposed by Arditti [46]. Stage 0 was no germination; Stage 1 was germination, in which the embryo swells and turns green, and the testa pops open; Stage 2 was protocorm formation, in which the embryo continuously enlarges and forms a spherule with the seed coat broken; Stage 3 was protocorm differentiation, indicated by the appearance of the protomeristem; Stage 4 was seedling formation, indicated by the emergence of the first leaf; and Stage 5 was seedling development, in which the second leaf emerges. Stages 0, 1, 2 + 3, and 4 + 5 were used to determine no germination, seed germination, protocorm formation, and seedling development, respectively. Total seeds (*t*), germinated seeds (*g*), protocorms (*p*), and seedlings (*s*) were counted at the time points for each treatment of the three different incubation experiments. The percentages of germinated seeds (*G*), protocorms (*P*), and seedlings (*S*) were calculated as follows: *G* = 100 × (*g* + *p* + *s*)/*t*, *P* = 100 × *(p+s)/t*, and *S* = 100 × *s*/*t*, respectively [14].

The effects of different fungal inoculations on seed germination, protocorm formation, and seedling development were compared using either one-way ANOVA and least significant difference (*LSD*) when data were subjected to normal distribution or generalized linear models (*GLMs*) when data did not meet normal distribution. A two-way ANOVA test followed by multiple comparison tests was performed to detect the differences between seed pretreatment times and different fungi under the three different fungal treatment conditions. All statistical analyses were performed in SPSS (version 25.0). The results are expressed as the mean ± standard error (mean ± SE), and the alpha-type I error was fixed at 5% (thus, all nonsignificant differences were determined based on *P* > 0.05).

## Supplementary Information

**Additional file 1:** **Table S1.** The percentages of seed germination, protocorm formation and seedling development at 52 days after incubation with two compatible fungi TPYD-2 and PI and one incompatible fungus FDd1. For each fungal incubation treatment, seeds of *Dendrobium officinale* were pretreated with 1% (w/v) NaClO for 0, 5, 10, 20, 30 and 40 min, respectively. In each fungal incubation treatment, different letters indicate significant differences based on one-way ANOVA and the least significant difference (*LSD*) method where the data compliant with normal distribution, and the generalized linear model (*GLM*) where the data is not normal distributed, respectively.

**Additional file 2:** **Figure S1.** The protocorms of *Dendrobium officinale*, that were produced by in vitro-seed germination on MS medium for 22 days to Stage 2, were used to assess the effects of seed coat removal on seed germination and seedling formation among different compatible and incompatible fungi treatments. The red arrow indicates the seed coat being removed completely.

## Data Availability

The datasets used and/or analyzed during the current study are available from the corresponding author on reasonable request.
