# Supplementary material for: What role does the seed coat play during symbiotic seed germination in orchids: an experimental approach with Dendrobium officinale
Source: BMC Plant Biol. 2022 Jul 29;22:375. doi: 10.1186/s12870-022-03760-0 (PMC9336064; doi:10.1186/s12870-022-03760-0)
Supplement: Supplementary file 1 — Additional file 1: Table S1. The percentages of seed germination, protocorm formation and seedling development at 52 days after incubation with two compatible fungi TPYD-2 and PI and one incompatible fungus FDd1. For each fungal incubation treatment, seeds of Dendrobium officinale were pretreated with 1% (w/v) NaClO for 0, 5, 10, 20, 30 and 40 min, respectively. In each fungal incubation treatment, different letters indicate significant differences based on one-way ANOVA and the least significant difference (LSD) method where the data compliant with normal distribution, and the generalized linear model (GLM) where the data is not normal distributed, respectively. [file 12870_2022_3760_MOESM1_ESM.docx]

**Appendix Table S1**. The percentages of seed germination, protocorm formation and seedling development at 52 days after incubation with two compatible fungi TPYD-2 and PI and one incompatible fungus FDd1. For each fungal incubation treatment, seeds of *Dendrobium officinale* were pretreated with 1% (w/v) NaClO for 0, 5, 10, 20, 30 and 40 min, respectively. In each fungal incubation treatment, different letters indicate significant differences based on one-way ANOVA and the least significant difference (*LSD*) method where the data compliant with normal distribution, and the generalized linear model (*GLM*) where the data is not normal distributed, respectively.

| **Seed pretreatment time** | **Seed germination (%)** | | |  | **Protocorms formation (%)** | | |  | **Seedlings formation (%)** | | |
| --- | --- | --- | --- | --- | --- | --- | --- | --- | --- | --- | --- |
|  | TPYD-2 | PI | FDd-1 |  | TPYD-2 | PI | FDd-1 |  | TPYD-2 | PI | FDd-1 |
| 0 min | 91.5±1.68ab | 83.4±2.48b | 78.3±3.24b |  | 89.5±1.73ab | 81.5±2.78b | 63.8±5.71b |  | 23.9±2.81a | 39.2±4.98d | ng |
| 5 min | 95.2±1.53a | 98.3±0.74a | 84.5±3.37ab |  | 95.2±1.53a | 98.3±0.74a | 75.9±5.77a |  | 26.2±3.4a | 80.0±2.21a | ng |
| 10 min | 87.8±4.66ab | 85.9±1.84b | 87.2±1.76a |  | 84.9±5.24bc | 83.2±3.74b | 87.0±1.77a |  | 27.9±4.67a | 64.0±5.68b | ng |
| 20 min | 85.7±3.98bc | 86.7±2.10b | 82.7±1.58ab |  | 88.1±2.95ab | 81.4±3.71b | 79.9±1.67a |  | 5.8±1.51b | 50.2±6.1bcd | ng |
| 30 min | 78.2±2.14cd | 86.1±1.85b | 67.2±3.00c |  | 77.3±2.31cd | 86.3±1.84b | 60.9±4.18b |  | 28.9±5.15a | 56.4±6.23bc | ng |
| 40 min | 70.1±3.05d | 72.4±3.12c | 62.4±3.22c |  | 69.7±3.1d | 71.1±3.57c | 60.5±3.54b |  | 21.7±3.69a | 45.4±5.39cd | ng |
