# Supplementary figures and images for: What role does the seed coat play during symbiotic seed germination in orchids: an experimental approach with Dendrobium officinale
Source: BMC Plant Biol. 2022 Jul 29;22:375. doi: 10.1186/s12870-022-03760-0 (PMC9336064; doi:10.1186/s12870-022-03760-0)

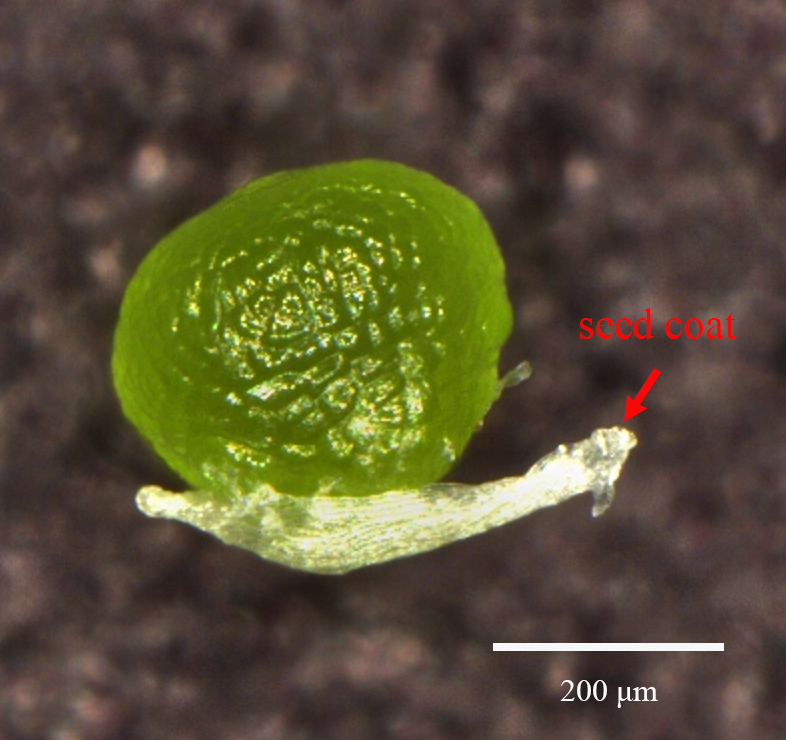

Supplement: Supplementary file 2 — Additional file 2: Figure S1. The protocorms of Dendrobium officinale, that were produced by in vitro-seed germination on MS medium for 22 days to Stage 2, were used to assess the effects of seed coat removal on seed germination and seedling formation among different compatible and incompatible fungi treatments. The red arrow indicates the seed coat being removed completely. [file 12870_2022_3760_MOESM2_ESM.tif]
